# Childbearing desire and reproductive behaviors among women living with HIV: A cross-sectional study in Abidjan, Côte d’Ivoire

**DOI:** 10.1371/journal.pone.0239859

**Published:** 2020-10-21

**Authors:** Shino Arikawa, Patricia Dumazert, Eugène Messou, Juan Burgos-Soto, Thierry Tiendrebeogo, Angèle Zahui, Apollinaire Horo, Albert Minga, Renaud Becquet

**Affiliations:** 1 Inserm, UMR 1219, Bordeaux Population Health Research Center, Team IDLIC, French National Research Institute for Sustainable Development (IRD), University of Bordeaux, Bordeaux, France; 2 Programme PAC-CI, ANRS site in Côte d’Ivoire, Centre Hospitalier Universitaire de Treichville, Abidjan, Côte d'Ivoire; 3 Centre de Prise en charge de Recherche et de Formation (CePReF-Aconda-VS), Abidjan, Côte d'Ivoire; 4 Service de gynécologie obstétrique, Centre Hospitalier Universitaire de Yopougon, Abidjan, Côte d’Ivoire; 5 Centre Médical de Suivi de Donneurs de Sang (CMSDS), Abidjan, Côte d’Ivoire; University of Botswana, BOTSWANA

## Abstract

**Introduction:**

Evidence on childbearing desire and reproductive behaviors in women living with HIV on antiretroviral therapy (ART) is scarce, particularly in West Africa. We investigated the prevalence and associated factors of childbearing desire in HIV-infected women in care in Abidjan, Côte d’Ivoire and explored whether such desires were translated into behaviors related to contraceptive use and communication with health personnel.

**Methods:**

A cross-sectional survey was conducted in two HIV-care facilities in Abidjan, Côte d’Ivoire in 2015. Eligible women were non-pregnant, non-menopausal, aged 18–49 years and diagnosed as HIV-infected. The outcomes were childbearing desire, prevalence of modern contraceptive use, unmet needs for family planning and intention of the last pregnancy since HIV diagnosis. Women wishing to conceive immediately were asked whether they had discussed their desire with HIV healthcare workers. Logistic regression models were used to assess the associations between the outcomes and women’s characteristics.

**Results:**

Of 1,631 women, 80% declared having childbearing desire. No association was found between women’s childbearing desire and ART status or its duration. In multivariate models, younger age, being in a stable relationship and having no or only one child were significantly associated with increased childbearing desire. Of the women wishing to conceive immediately (n = 713), only 43% reported having had fertility-related dialogue with healthcare provider. Among sexually active women wanting to avoid or delay pregnancy (n = 650), unmet needs for family planning was 40%. Regarding the last pregnancy since HIV diagnosis, one in three women reported not having wanted a baby at that time.

**Conclusions:**

Pregnancy desire in women living with HIV in Abidjan was extremely high. Integration of safe conception strategies as well as improvement of contraceptive uptake among women in need of family planning are of utmost importance to ensure optimal conception and to avoid transmission of HIV to the male partner or to the forthcoming child.

## Introduction

Improved access to prevention of mother-to-child transmission of HIV (PMTCT) services and antiretroviral therapy (ART) have dramatically changed reproductive choice in women living with HIV. In the pre-ART era, HIV-infected women were often considered as not fit to become mother due to ill health, compromised childcare capacity and survival, and most importantly because of the risk associated with the vertical transmission of HIV [1]. Despite such stigmatizing views, studies have shown that HIV-infected women had childbearing desires and pursued reproductive goals to have their own biological child [1–3]. Societal expectation on woman’s role as mother and cultural values related to large family, especially in the context of sub-Saharan Africa, motivated women to conform to the norm despite potential risks [3].

With the protection of ART, HIV transmission risks, both vertically and horizontally, are extremely low [4–6]. One might think that HIV-infected women on ART would feel more assured thus encouraged to conceive and by consequence have increased desire for motherhood [7]. While studies from Europe and North America generally confirm such hypothesis [8], evidence from sub-Saharan Africa is very limited and, if any, largely comes from Eastern and Southern Africa [2, 9–12]. The results of these studies are not conclusive however, showing increased desire in women on ART in some settings [2, 9, 10], but not elsewhere [11, 12].

HIV care, regardless where it is located, should be able to respond adequately to different client’s needs, whether for optimal conception or for limiting or avoiding pregnancies. In recent years, several approaches for safe conception have been proposed, including reduction of women’s infectiousness through ART, use of pre-exposure prophylaxis by non-infected partner, sperm washing, artificial insemination, unprotected sex during fertile period and voluntary male circumcision [13]; however, access to and availability of such services are limited in many resource-limited settings [14, 15]. It might not be surprising therefore that prevalence of unplanned pregnancies and unmet needs for family planning in HIV-infected women are still alarmingly high, raising concerns for persisting risk of vertical and horizontal HIV transmission and of adverse effects on women’s own health [16–18].

Côte d’Ivoire is one of the countries most affected by HIV in West Africa, with a prevalence of 2.7% [19]. Women account for more than a half of adults (≥15 years) living with HIV in the country [19]. While the country intensifies its effort to improve PMTCT and ART coverage with extended eligibility [20], integration of reproductive health services in HIV care has not reached to sufficient level, let alone implementation of client-oriented service delivery related to safe conception and prevention of unplanned pregnancy.

The present study aims to assess the prevalence and determinants of childbearing desire in HIV-infected women in care in Abidjan, Côte d’Ivoire. We also explored if their desires were translated into behaviors related to contraceptive use and communication with health personnel. Finally, we assessed if pregnancies that had occurred after HIV diagnosis were planned and estimated the frequency of unintended pregnancies in this population.

## Methods

### Study design and setting

We conducted in 2015 a cross-sectional survey in two HIV-care facilities in Abidjan, Côte d’Ivoire, located in the most densely populated areas of the city, Treichville and Yopougon. These secondary-level facilities offer to patients routine clinical visits every three months with free of charge provision of ART. ART eligibility at the time of the study was CD4 count below 350 cells/mm^3^. Reproductive health services were not routinely offered in any of these facilities, and patients were referred to counselors for family planning issues, either upon explicit request from patients themselves or clinical call made by health workers. Provision of male condoms was free and unrestricted on patients’ demand. Other contraceptive commodities including implant, intrauterine device (IUD), oral contraception pills and related-services were not available in these facilities, and patients were referred to services dedicated for family planning. No particular safe conception strategy was put in place at the time of the survey. In the communities surrounding these facilities, male condoms are easily accessible in pharmacies and other small shops.

### Study population

Eligible women were non-pregnant, pre-menopausal, aged between 18–49 years, and diagnosed as HIV positive. Women were approached individually while waiting for their routine HIV medical appointment and informed of the purpose of the study. Only those agreeing to participate underwent informed consent process and completed the survey administered by trained midwives in a private space.

### Data collection

Information on socio-demographic characteristics of the women and their reproductive history were gathered. Women who declared being in a stable relationship were asked to provide information on their partner. Childbearing desire was defined by women’s response to the question “Would you like to have a child in the future?” (yes, no, I don’t know). When women answered affirmatively, they were asked to choose a desired timing of conception, either immediately, in 1–2 years, or sometime in the future. The questionnaire was pre-tested and modified as necessary.

The intention of the last pregnancy since the diagnosis of HIV status was assessed using the London Measure of Unplanned Pregnancy (LMPU) [21]. The LMPU is a psychometrically-validated measure of the degree of intention of a current or recent pregnancy, constituted by six questions rated 0, 1 or 2, providing a final score ranging from 0 to 12. We asked women a series of pre-defined questions related to their feelings about the pregnancy and how they prepared for it. At the time of the survey, there was no validated French version of the LMPU, thus the study team undertook the translation work. The translated version was tested with local midwives in order to fully ensure comprehension of the study questions.

### Statistical analysis

Characteristics of the women were described as median and frequency for continuous and categorical variables, respectively. Women’s childbearing desire was classified into two distinct categories, grouping together those having no or ambiguous desire, which was compared to those reporting positive childbearing desire. Logistic regression models were used to estimate the association between women’s childbearing desire and the following factors: ART (yes/no), age (18–24, 25–29, 30–34, 35–39, 40–44), education (no formal education, primary, secondary, tertiary or above), remunerated activity, presence of a male partner, HIV status disclosure to partner, and the number of living children (0, 1, ≥2). Additionally, women’s ART status was modelled in several different manners to see if the duration of ART (30, 180 or 365 days since ART initiation) was associated with childbearing desires. To investigate if couple-related characteristics influence women’s childbearing desire, we also undertook sub-analysis among women in a relationship. Missing data were included as a separate category to maintain sample size. Explanatory variables were selected on the basis of prior knowledge on associated factors. We used stepwise-descending approach to identify association. In the final model, statistical significance was reached when P-value <0.05.

Behaviors of women were also explored according to their respective pregnancy desire. Among those sexually active and wishing to delay or avoid pregnancy, we assessed the prevalence of modern contraceptive use to see if their behaviors correspond with their desire to limit pregnancies. Among women wishing to conceive immediately, we estimated the proportion of those who had discussed such childbearing intentions with health personnel.

The intention of the last pregnancy since HIV diagnosis was assessed based on the LMPU score and the criteria recommended by Barrett et al. [21]. A score between 0–3, 4–9 or 10–12 meant that the pregnancy was unplanned, ambivalent or planned, respectively. The distribution of pregnancy intention was described as frequency. This analysis was undertaken exclusively among women who had at least one pregnancy after their HIV diagnosis and who reported both the date of HIV diagnosis and of their last pregnancy in order to ensure temporality of the events.

### Ethics statement

This study was granted ethical permission in Côte d’Ivoire from the ethical committee of the National AIDS Control Programme, under the International epidemiology Database to Evaluate AIDS (IeDEA) West Africa collaboration. All study participants were informed of the objective of the study and provided written consent.

## Results

### Description of the study population

**Fig 1:**
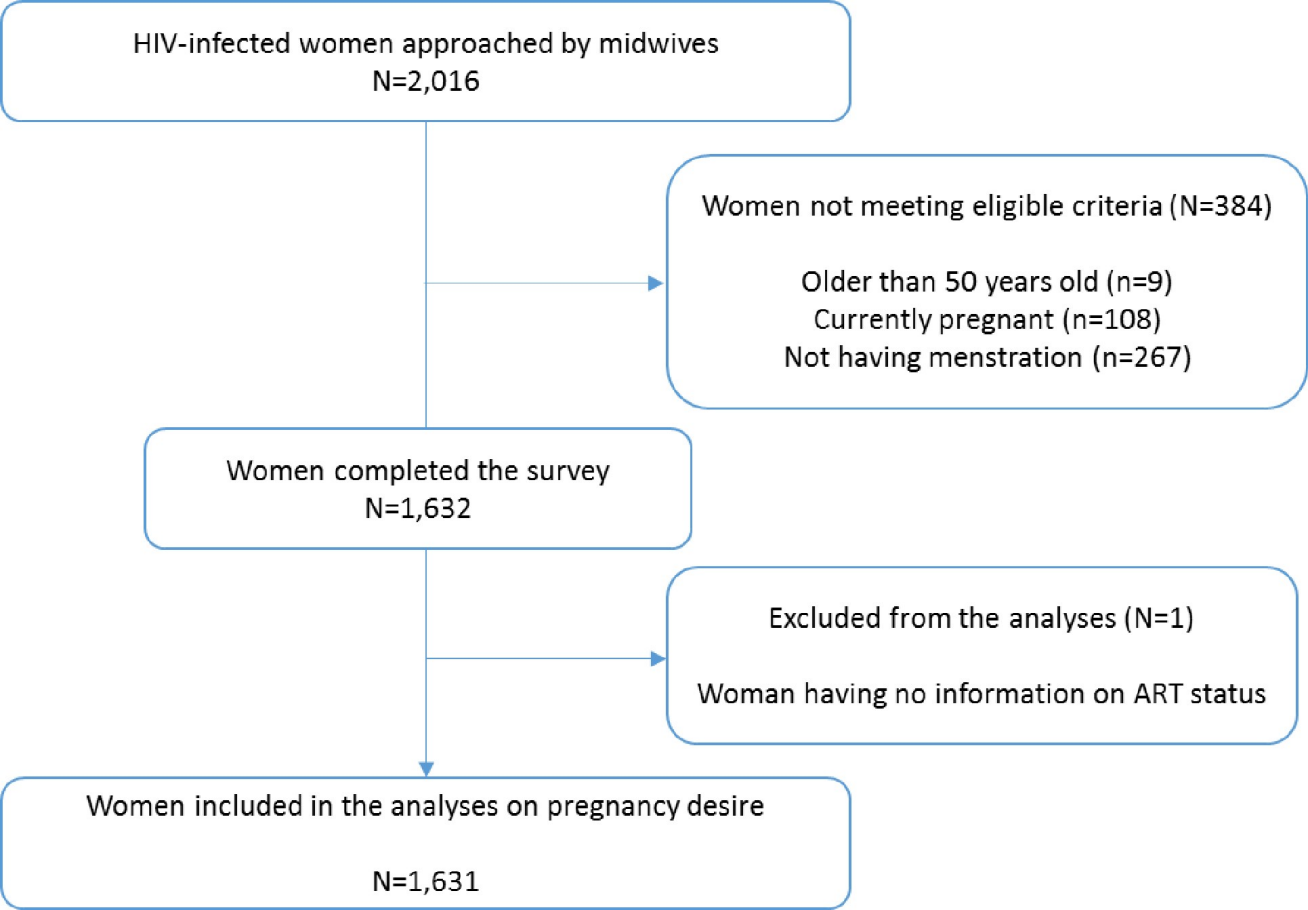
Flow chart on the selection of study population.

Of the 2,016 women approached by the study investigators, 1,632 met inclusion criteria and completed the survey. One woman was excluded from the analyses for not having information on ART (Fig 1).

The socio-demographic and reproductive health related characteristics of the 1,631 women included in the analyses are shown in Table 1. Median age was 37.0 years (Interquartile range (IQR): 32.4–41.5), almost a half achieved secondary or higher education, and 66% had a paid work. They been living with HIV for a median of 5.1 years (IQR: 2.2–8.3), and nearly 90% were on ART for a median duration of 52.9 months (IQR: 22.9–82.1). One quarter had no living child, 29% had one and 44% had two or more. Women had a median of three lifetime pregnancies (IQR: 2–5). Overall, 75% (n = 1,230) were in a relationship. Of these, 21% had a partner known to be HIV positive (n = 253), and 32% had a partner having been tested negative for HIV (n = 392). The remaining 47% (n = 585) did not know the HIV status of their partner.

**Table 1:** Description of the study population (N = 1,631).

	N	%
**Age**		
18–24	54	3.3
25–29	164	10.1
30–34	379	23.2
35–39	483	29.6
Over 40	551	33.8
**Education**		
No formal education	360	22.1
Primary	496	30.4
Secondary	550	33.7
Tertiary or above	225	13.8
**Having paid work**		
No	553	33.9
Yes	1,078	66.1
**On ART**		
No	201	12.3
Yes	1,430	87.7
**Number of living children**		
0	437	26.8
1	478	29.3
≥ 2	715	43.8
Unknown	1	0.1
**In a relationship**		
No	401	24.6
Yes	1,230	75.4
**Contraceptive use (modern methods including male condoms)**		
No	804	49.3
Yes	827	50.7
**Contraceptive use (modern methods** ***not*** **including male condoms)**		
No	1,146	70.3
Yes	485	29.7
**Type of contraceptives used (n = 827)**		
Oral hormonal pills	407	49.2
Male condoms	342	41.4
Injectable	66	8.0
Intra-uterine device	9	1.1
Implant	3	0.3

Overall prevalence of modern contraceptives was 51% (n = 827). The majority were using short-term contraceptives including oral hormonal pills (49%, n = 407) and male condoms (41%, n = 342). Utilization of long-acting reversible contraception was extremely low: 8% for injectable (66/827), 1% for intrauterine device (IUD) (9/827), and 0.4% for implant (3/827). Of those who declared using male condoms or oral hormonal pills, 21% and 17% expressed dissatisfaction about the method, respectively. The rate was lower among women using injectable (10%), IUD (0%) or implant (0%).

### Childbearing desire and associated factors

Of 1,631 women included in the analyses, 80% expressed childbearing desire (n = 1,310), and 16% reported not wanting to have a child in the future (n = 255). The remaining (4%, n = 66) were not sure of their desire. Among 1,310 women who expressed explicit desire for childbearing, 54% (n = 713) wished to conceive immediately, 16% (n = 208) in one or two year and 30% (n = 389) sometime in the future.

In univariate analyses, women’s ART status was not associated with childbearing desire (P = 0.50). Similarly, there was no association between duration of ART and childbearing desire regardless of the thresholds used (30, 180 or 365 days since ART initiation). No association was found between women’s childbearing desire and work status or income level. Younger women were significantly more likely to report childbearing desire compared to those over 40 years of age (OR: 6.8, 5.8, 5.8, and 2.5 for 18–24, 25–29, 30–34, and 35–39 years age categories, respectively). Number of living children was inversely associated with increased childbearing desire. Women having tertiary or higher education were significantly more likely to report having childbearing desire in univariate analysis, however this association became statistically non-significant when adjusting for study site, age, and number of living children. Adjusted for study site and women’s socio-economic characteristics (education, age, work, relationship status), there was no association between women’s ART status and childbearing desire. Younger age, being in a relationship (married or other types of relationship), having no or only one child, and having a paid work were significantly associated with increased childbearing desire in multivariate model (Table 2).

**Table 2:** Associated factors with childbearing desire among women living with HIV (N = 1,631).

	Having childbearing desire		Having no childbearing desire		Univariable				Multivariable			
	(N = 1,310)		(N = 321)									
	n	%	n	%	OR	95%CI		P	aOR	95%CI		P
**Study site**	** **	** **	** **	** **	** **	** **	** **	< .0001	** **	** **	** **	< .0001
Treichville	599	74.6	204	25.4	1	** **	** **	** **	1	** **	** **	** **
Yopougon	711	85.9	117	14.1	2.07	1.61	2.66	** **	2.61	1.95	3.50	** **
**Age category (in years)**	** **	** **	** **	** **				< .0001				< .0001
18–24	51	94.4	3	5.6	8.59	2.65	27.90		3.95	1.15	13.53	
25–29	151	92.1	13	7.9	5.87	3.24	10.63		3.89	2.05	7.38	
30–34	348	91.8	31	8.2	5.67	3.77	8.53		4.51	2. 90	7.02	
35–39	394	81.6	89	18.4	2.24	1.67	2.99		2.02	1.46	2.81	
40–49	366	66.4	185	33.6	1				1			
**Education**	** **	** **	** **	** **				0.05				0.29
No formal education	281	78.1	79	21.9	1				1			
Primary	397	80.0	99	20.0	1.13	0.80	1.57		1.19	0.81	1.75	
Secondary	436	79.3	114	20.7	1.08	0.78	1.49		0.85	0.58	1.24	
Tertiary or above	196	87.1	29	12.9	1.90	1.20	3.02		0.92	0.53	1.58	
**Having a paid work**	** **	** **	** **	** **				0.14				0.02
No	433	78.3	120	21.7	1				1			
Yes	877	81.3	201	18.7	1.21	0.94	1.56		1.41	1.05	1.90	
**Monthly income**	** **							0.22				
less than 90 USD	932	80.3	228	19.7	1							
90–180 USD	195	83.7	38	16.3	1.26	0.86	1.83					
above 180 USD	159	77.9	45	22.1	0.86	0.60	1.24					
Unknown	24	70.6	10	29.4	0.59	0.28	1.25					
**On ART**	** **	** **	** **	** **				0.50				0.70
No	165	82.1	36	17.9	1				1			
Yes	1145	80.1	285	19.9	0.88	0.60	1.29		1.10	0.70	1.73	
**No. of living children**	** **	** **	** **	** **				< .0001				< .0001
0	413	94.5	24	5.5	1				1			
1	433	90.6	45	9.4	0.56	0.34	0.93		0.53	0.31	0.90	
≥ 2	463	64.8	252	35.2	0.11	0.07	0.17		0.10	0.06	0.17	
Unknown	1	100.0	0	0	*				*			
**Relationship status**	** **	** **	** **	** **				< .0001				< .0001
Single	279	70.3	118	29.7	1				1			
Married	288	74.2	100	25.8	1.22	0.89	1.66		1.78	1.23	2.57	
Other types of relationship	739	12.2	103	87.8	3.03	2.25	4.09		2.79	1.97	3.95	
Unknown	4	100.0	0	0	*				*			

*Not estimated due to small number of patients under this category.

To investigate the impact of couple-related characteristics on women’s childbearing desire, we undertook a sub-analysis on the women who have a partner (n = 1,230). As Table 3 shows, unmarried women, whether living with partner or not, were significantly more likely than married women to report childbearing desire in univariate analysis (P < .0001). Women whose partner was known to be HIV-infected were significantly less likely to report their desire to have a child compared to women whose partner was not known to be HIV positive (OR: 0.46, 95%CI: 0.34–0.62). Likewise, women who declared having disclosed their HIV status to their partner were significantly less likely to report having childbearing desire. However, once women’s age, number of living children and study sites were allowed in multivariate analyses, HIV status of partner and disclosure variable were no longer associated. Lack of association with these variables were also confirmed by several sensitivity analyses using different age categories (binary 18–29 vs. over 30 years; three categories 18–24, 25–34 and over 35 years). In multivariate analyses, unmarried women who were not living with the partner were significantly more likely to report childbearing desire than married women.

**Table 3:** Associated factors with childbearing desire in women living with HIV who have a partner (N = 1,230).

	Having childbearing desire		Having no childbearing desire		Univariable				Multivariable*			
	(N = 1,027)		(N = 203)									
	n	%	n	%	OR	95%CI		P	aOR	95%CI		P
**Type of relationship**	** **	** **	** **	** **	** **	** **	** **	< .0001	** **	** **	** **	0.047
Married, living together	288	74.2	100	25.8	1	** **	** **	** **	1	** **	** **	** **
Not married, living together	415	87.2	61	12.8	2.36	1.66	3.36	** **	1.43	0.96	2.12	** **
Not married, not living together	324	88.5	42	11.5	2.68	1.80	3.97	** **	1.66	1.07	2.58	** **
**Partner's HIV status**								< .0001				
Positive	186	73.5	67	26.5	1							
Negative	328	83.7	64	16.3	1.85	1.25	2.72					
Unknown	513	87.7	72	12.3	2.57	1.77	3.73					
**Woman's HIV status disclosed to partner**								0.001				
No	392	88.1	53	11.9	1							
Yes	629	80.7	150	19.3	0.57	0.40	0.80					
Unknown	6	100.0	0	0	**							

*Adjusted for woman's age category, number of living children and study site.

**Not to be estimated due to small number.

### Dialogue with health personnel and unmet family planning needs

Of the 713 women who reported having a desire to conceive immediately, only 43% declared having discussed their fertility desire with health personnel (297/713). Dialogue with health personnel was also infrequent among 650 women who were sexually active and wanted to delay or to avoid pregnancy: only 12% (55/650) declared having consulted health personnel on family planning options. Unmet family planning needs were high among these women: 40% (258/650) reported not using any types of modern contraceptive methods.

### Intention of the last pregnancy since HIV diagnosis

**Fig 2:**
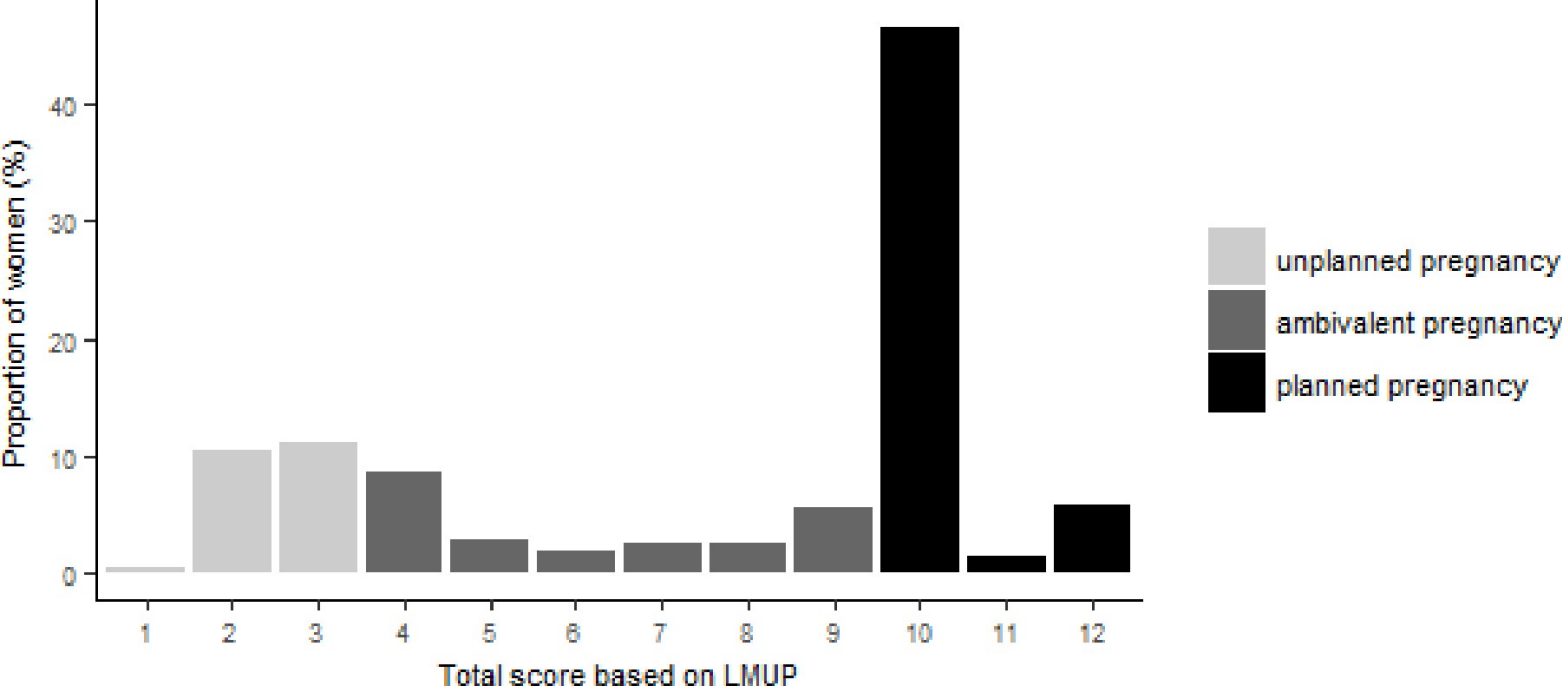
Distribution of overall score based on the London Measure of Unplanned Pregnancy (N = 480).

A total of 481 women were included in the assessment of intention on the last pregnancy since HIV diagnosis. As Fig 2 shows, the overall LMUP scores were distributed fairly unevenly with high concentration on the score 10 (47.5%), and the remaining on the score 3 (11.3%), 2 (10.4%), 4 (8.5%), 12 (5.8%), and 9 (5.6%).

Based on the LMUP definition, 54% of the last pregnancies were considered as planned (score 10–12), 24% as ambivalent (score 4–9), and 22% as unplanned (score 0–3). Women’s responses to specific items of the questionnaire are provided in Table 4.

**Table 4:** Intention of the last pregnancy since HIV diagnosis using the London Measure of Unplanned Pregnancy.

	N	%
**In the month that I became pregnant…**		
I was not using contraception in all	444	92.5
I was using contraception, but not every time we had sex	31	6.5
I always used contraception, but knew that the method had failed at least once	5	1.0
**In terms of becoming a mother, I feel that my last pregnancy happened at the…**		
Right time	291	60.6
Ok, but not quite right time	90	18.8
Wrong time	99	20.6
**Just before I became pregnant this time…**		
I intended to get pregnant	309	64.4
My intentions kept changing	3	0.6
I did not intend to get pregnant	168	35.0
**Just before I became pregnant this time…**		
I wanted to have a baby	314	65.4
I had mixed feelings about having a baby	12	2.5
I did not want to have a baby	154	32.1
**Before I became pregnant…**		
My partner and I had agreed that we would like me to be pregnant	300	62.5
My partner and I discussed having children together, but hadn't agreed for me to get pregnant	79	16.5
We never discussed having children together	101	21.0
**Before you became pregnant, did you do anything in preparation for pregnancy?**		
Yes, I took one or more following actions (multiple response allowed):	47	9.8
*Took folic acid*	*5 *	
*Stopped or cut down smoking*	*0 *	
*Stopped or cut down drinking alcohol*	*2 *	
*Ate more healthily*	*0 *	
*Got medical advice*	*28 *	
*Did something else to prepare*	*14 *	
No, I did not do any of the above before my pregnancy	433	90.2
**LMUP Score**		
Unplanned pregnancy (score 0–3)	107	22.3
Ambivalent pregnancy (score 4–9)	115	24.0
Planned pregnancy (score 10–12)	258	53.7

The great majority (93%) reported not having been using any contraceptives during the month they became pregnant. Overall, 61% said that their pregnancy happened at the right time and 64% actually intended to get pregnant. One in three women reported not having wanted a baby at that time. Most women (90%) did not take any particular actions as preparation for conception while a small number of women sought medical advice (6%) or took other initiatives (3%) including purchase of traditional medicine.

## Discussion

Our study showed that more than 80% of HIV-infected women desired children, a rate particularly high compared to what was reported from other HIV populations in sub-Saharan Africa: 29% in Uganda [11], 30% in Kenya [22], 26% to 31% in South Africa [2, 12]. This high prevalence of childbearing desire in our study clearly highlights the extent to which women valued childbearing in Abidjan. The reported rate is fairly striking given high median age in our study population (37 years) and that they already had three lifetime pregnancies. This high median age and the fact that the women in a relationship (but not married) had the highest childbearing desire could suggest that these women were married once and are actually in a new relationship where they feel the need to prove their fertility. Unfortunately, we could not verify this hypothesis as we did not have any information on women’s marital status in the past. Desire for children among HIV-infected women was associated with younger age, being in a relationship, and fewer number of living children. Childbearing desire was not different according to ART status or the duration of ART. Similarly, there was no association with HIV status of the partner or whether or not women’s HIV status had been disclosed to the male partner. Fertility rate in Côte d’Ivoire is one of the highest in the world with a total fertility rate of five children per woman (3.1 in Abidjan), and more than 70% of women in reproductive age in Côte d’Ivoire wished to have a (or another) child in the future [23]. Motherhood and large family are highly valued and interpreted as wealth and security for the family. In such societal context, HIV and ART-related factors might not provide full explanation on the motivation behind fertility decision in women or couples affected by HIV. While our data does not allow to comment on whether or not this high level of childbearing desire is observed in HIV-uninfected counterparts, it clearly highlights the needs for better integration of reproductive health services into HIV care and the importance of making these services more client-oriented. In this context, lack of clear strategies on safe conception in the current HIV care should be seen as a gap between client’s needs and existing services. We also showed that the vast majority of women in need of counselling for immediate conception did not seek any advice from health personnel. Health-care providers should initiate dialogue on pregnancy intentions in order to ensure that sufficient precautions are taken to avoid vertical and horizontal transmission of HIV and to fully achieve reproductive rights of women living with HIV.

Furthermore, high unmet family planning needs and the reluctance to seek advice on contraception were also clearly shown by our study. Although a half of women declared using modern contraceptives, many used short-term solutions such as male condoms. Due to low effectiveness of barrier methods in preventing unwanted pregnancy [24], healthcare providers should be reminded that such method should be kept as one of many options and promoted solely as a component of method mix. Available evidence does not suggest that women living with HIV using ART should have restrictions on the choice of hormonal contraceptives, and most contraceptive methods are rated as Medical Eligibility Criteria 2 (A condition where the advantages of using the method generally outweigh the theoretical or proven risks: GRADE moderate to very low quality of evidence) by the World Health Organization, except for IUD for women with advanced HIV stage due to potential risk of pelvic inflammatory disease following IUD placement [25–27]. Women wishing to avoid or delay pregnancy should therefore receive information and counselling on the whole range of contraceptive methods and be encouraged to make informative decision. Despite regular contacts with health personnel, HIV-infected women have rarely sought advice from health personnel on family planning. Their reluctance might be related to partner’s desire for having more children, his unwillingness for the woman to use contraceptive, lack of decision making or negotiating power on women’s part, or perceptions related to contraception and family planning in general. The most recent Demographic Health Survey in Côte d’Ivoire reported a low prevalence of modern contraceptive methods in Abidjan in the general population (21%), which suggests cultural or societal factors influencing low acceptability [23]. Quality of messages from health personnel might not be fully adapted to untangle certain belief or doubts on contraceptives. An in-depth socio-anthropologic approach would be needed to identify underlying causes of persisting low contraceptive use in HIV-infected women who wish to avoid or delay pregnancies.

Our study also shed light on pregnancy intention in HIV-infected women after HIV diagnosis. While about a half of most recent pregnancies were considered as intended, caution is needed in interpreting this result due to limitations in the method used. In the LMUP, non-utilization of contraceptives was weighted towards having a pregnancy intention, however such a simple interpretation can be misleading in a place like Côte d’Ivoire where motherhood and childbearing are highly appreciated, and misconceptions on contraceptives are still widely held. In our study, the majority of women considered as having had pregnancy intention (86%, 223/258) scored 10 in the LMUP, the lowest score for being qualified as having a planned pregnancy. Of these, 99% (221/223) were not using any contraceptives. These pregnancies would not have been defined as ambivalent if we were to assign less weight on the utilization of contraceptives as an indicator for pregnancy intention. This means that the number of pregnancies that could be defined as planned is indeed very few.

Our study has several limitations. Pregnancy desire and intention are notions extremely complex to capture and might not be adapted for binary classification. The cross-cutting nature of the study also hinders full understanding of the notions which are far from static and subject to temporal changes in personal and couple dynamics. Moreover, retrospective investigation on the previous pregnancy might have caused recall biases, and social desirability related childbearing could have played a part in certain responses of the women.

## Conclusions

Pregnancy desire in women living with HIV in Abidjan was extremely high. There is an urgent need to cater for different reproductive needs and desires of these women. Integration of safe conception strategies within the existing HIV care services, as well as improvement of contraceptive uptake among women in need for family planning are of utmost importance not only to avoid unplanned pregnancies but also to ensure an optimal timing for conception. The results of our study are useful for identifying policy priorities and interventions in order to fulfil reproductive health and rights of women living with HIV. As HIV is increasingly recognized as a chronic disease [3, 28], and with an increasing number of women initiating and continuing on ART, HIV care services should adequately respond to different childbearing desires of women living with HIV over their reproductive life span.
